# Chinese Medicine Formula Kai-Xin-San Ameliorates Neuronal Inflammation of CUMS-Induced Depression-like Mice and Reduces the Expressions of Inflammatory Factors via Inhibiting TLR4/IKK/NF-κB Pathways on BV2 Cells

**DOI:** 10.3389/fphar.2021.626949

**Published:** 2021-03-11

**Authors:** Suchen Qu, Mengqiu Liu, Cheng Cao, Chongqi Wei, Xue-Er Meng, Qianyin Lou, Bin Wang, Xuan Li, Yuyan She, Qingqing Wang, Zhichao Song, Zhengxiang Han, Yue Zhu, Fei Huang, Jin-Ao Duan

**Affiliations:** ^1^ Jiangsu Key Laboratory for High Technology Research of TCM Formulae and Jiangsu Collaborative Innovation Center of Chinese Medicinal Resources Industrialization, Nanjing University of Chinese Medicine, Nanjing, China; ^2^ Department of Neurology and Rehabilitation, Shanghai Seventh People’s Hospital, Shanghai University of TCM, Shanghai, China; ^3^ Department of Endocrinology, Suzhou TCM Hospital Affiliated to Nanjing University of Chinese Medicine, Suzhou, China

**Keywords:** Kai-Xin-San, neuronal inflammation, toll like receptor 4, NF-κB, Chinese medicine formulae

## Abstract

Kai-Xin-San (KXS) is a traditional Chinese medicinal formula composed of Ginseng Radix et Rhizoma, Polygalae Radix, Acori Tatarinowii Rhizoma, and Poria for relieving major depressive disorder and Alzheimer’s disease in traditional Chinese medicine (TCM) clinics. Previous studies on the antidepressant mechanism of KXS mainly focused on neurotransmitter and neurotrophic factor regulation, but few reports exist on neuronal inflammation regulation. In the current study, we found that KXS exerted antidepressant effects in chronic unpredictable mild stress-induced depression-like mice according to the results of behavioral tests. Meanwhile, KXS also inhibited the activation of microglia and significantly reduced the expression of pro-inflammatory cytokines such as IL-1β, IL−2, and TNF-α in the hippocampus of mice. In mice BV2 microglia cell lines, KXS extract reduced the expression of inflammatory factors in BV2 cells induced by lipopolysaccharide via inhibiting TLR4/IKK/NF-κB pathways, which was also validated by the treatment of signaling pathway inhibitors such as TAK-242 and JSH-23. T0hese data implied that the regulation of pro-inflammatory cytokines in microglia might account for the antidepressant effect of KXS, thereby providing more scientific information for the development of KXS as an alternative therapy for major depressive disorder.

## Introduction

Major depressive disorder (MDD), a widespread mental disorder characterized by the presence of sadness, pleasure loss, and somatic and cognitive changes, has become a serious public health problem (Malhi and Mann, 2018). Its pathogenesis is extremely complex, and various pathogenesis hypotheses, such as neurotransmitter imbalance, deficient supply of neurotrophic factors, and over-stimulation of the hypothalamus-pituitary gland-adrenal gland (HPA) axis, have been proposed (Otte et al., 2016). In recent years, an increasing number of studies have shown that chronic inflammation of the central nervous system (CNS) is crucial to the occurrence of depression. Patients with inflammatory autoimmune diseases such as multiple sclerosis, diabetes, and rheumatoid arthritis have been found to have a higher incidence of depression. The levels of interleukin-1 beta (IL-1β), interleukin-6 (IL-6), and tumor necrosis factor alpha (TNF-α) in the serum of patients with depression are also significantly higher than those of normal people (Pape et al., 2019). Animal studies also confirmed that persistent chronic stress activates the HPA axis, damages neurons in the hippocampus, and reduces the release of chemokine CX3CL1, which inhibits microglial activation. Microglia are principal immune cells located in the brain responsible for the upregulation of pro-inflammatory mediators upon activation, which is critical for the development of neuronal inflammation in the brain. Chronic overstimulation of microglia can lead to pro-inflammatory mediator release, including pathogenic proteins, cytokines, and chemokines, which significantly and adversely affect neurobiological structure and function. It has been clinically found that patients with depression have obvious signs of inflammation in the brain, accompanied by blood-brain barrier dysfunction. Concurrently, intracerebral necropsy of depressive suicide victims also revealed an abnormal increase in microglia density. Therefore, decreasing the levels of inflammatory cytokines in the brain and reducing neuronal inflammatory damage might be another target for the development of antidepressants (Dantzer, 2018).

Chinese medicine formulae are usually composed of several herbs, which can achieve synergistic effects through multiple targets. Kai-Xin-San (KXS) is comprised of four herbs, namely Ginseng Radix (GR), Polygalae Radix (PR), Acori Tatarinowii Rhizoma (ATR), and Poria (PO), and is used to relieve psychological diseases. According to the different symptoms of patients, the ratios of these four herbs (GR:PR:ATR:PO) are varied and three ratios are frequently used: 3:2:2:3 (D-652), 1:1:1:2 (K-984), and 1:1:4:8 (K-1640). The details can be found in Supplementary Table S1. At present, KXS is still used for treating MDD and Alzheimer's disease in clinical settings. In our previous studies, we found that KXS exerts antidepressant effects by increasing the supply of neurotransmitters and neurotrophic factors. In addition, KXS could exert an antidepressant effect by regulating the gut-brain axis, which included modification of gut microbiota distribution, suppression of the hypothalamus-pituitary-adrenal axis, and down-regulation of pro-inflammatory cytokines in the brain in chronic unpredictable mild stress (CUMS)-induced depression-like mice (Cao et al., 2018; Cao et al., 2020). In the current study, we aimed to elucidate the details and relationship between KXS exerting an antidepressant effect and suppressing the expression of pro-inflammatory cytokines in the brain. First, CUMS-induced depression-like mice were used as the animal model. Different compatible ratios of KXS were applied to treat the mice and behavioral tests were used to evaluate its antidepressant effect. In addition, the expression of pro-inflammatory cytokines in the brain and the status of microglia were determined. Second, KXS extracts were treated with lipopolysaccharide (LPS)-induced inflammatory mice BV2 microglia cell lines to evaluate the effect of suppressing pro-inflammatory cytokine expression, and the possible signaling pathway was explored. This study might be helpful for the elucidation of the antidepressant effect of KXS, which is beneficial for the development of KXS as an alternative therapy for patients with MDD.

## Materials and Methods

### Preparation of KXS Extract

**FIGURE 1:**
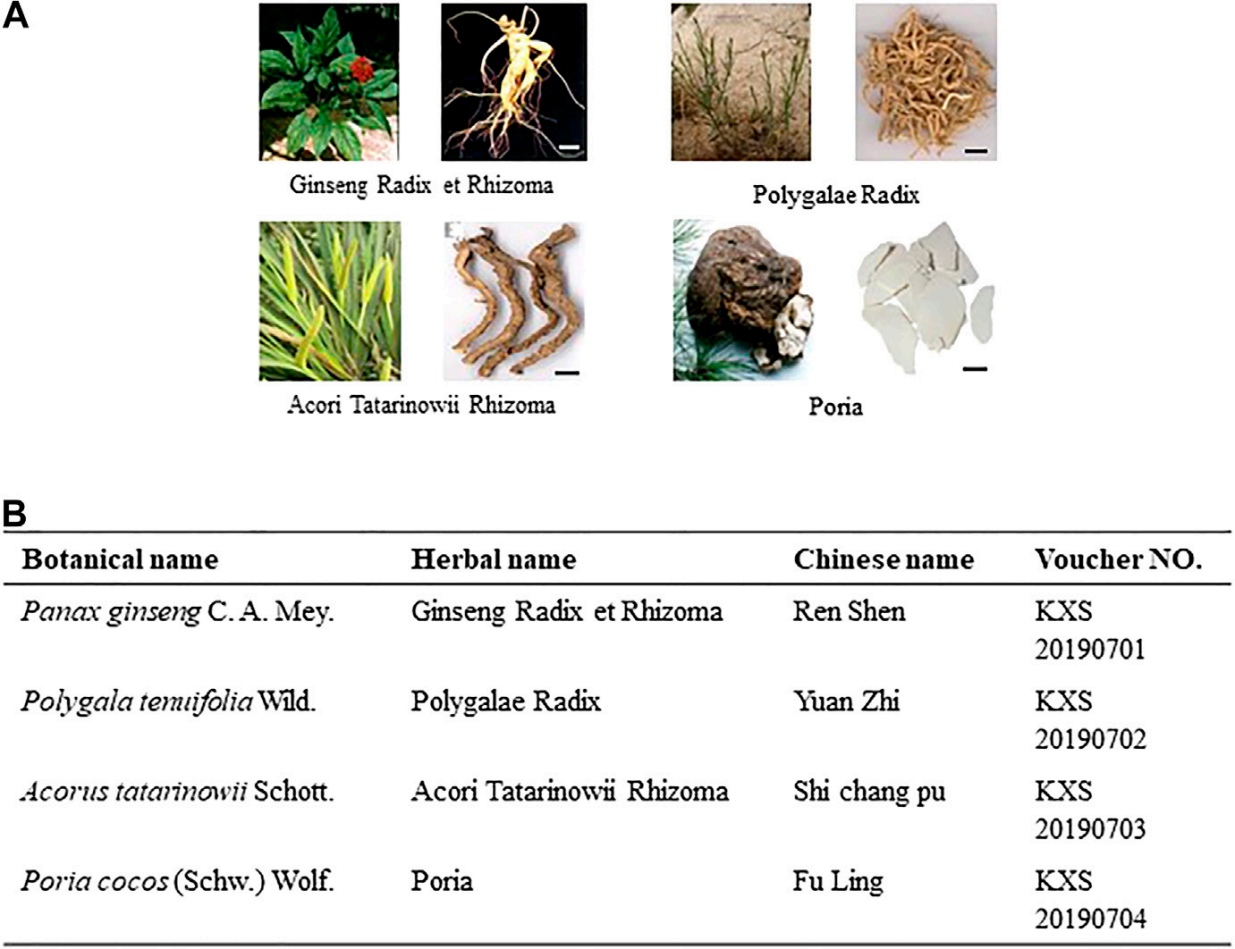
Information of components in KXS formula. **(A)**: Morphology of herbs in KXS. Bar = 1 cm. **(B)**: List of botanical, herbal, Chinese name, and voucher number of the corresponding herb in KXS.

The four herbs comprising KXS, namely Ginseng Radix, Polygalae Radix, Acori Tatarinowii Rhizoma, and Poria, were purchased from Suzhou Tianling Chinese Herbal Medicine Co. Ltd. They were identified as authentic medicinal materials by Prof. Hui Yan of Nanjing University of Chinese Medicine (NJUCM) according to their morphological characteristics. The details of the herbs are listed in Figure 1. According to the different compatibility ratios of KXS (Supplementary Table S1), the four herbs were mixed, weighing 100 g in total. They were soaked in 1,000 ml water, refluxed, and filtered. The residue was repeated for the same extraction procedures. The extraction solutions were combined and freeze-dried to obtain powders. The quality control procedures of KXS extracts can be found in our previous publication (Cao et al., 2020). The representative chromatograms of KXS extracts are displayed in Supplementary Figure S1, and the quantification results of the chemical amounts are listed in Supplementary Table S2.

### Animals and Housing Conditions

Male Institute of Cancer Research mice (body weight 22–25 g) were purchased from GemPharmatech (China). The animals were raised in the SPF environment of the experimental animal center of NJUCM). They were raised in a routine way, under a 12-h light/dark cycle, in a temperature of 22–25°C and a humidity of 40%–70%. The development of the CUMS animal model and behavioral and biochemical tests on the animals were approved by the Animal Experimental Ethics Committee of NJUCM and conformed to the guidelines of the “Public Health Service Policy on Human Care and Use of Laboratory Animals” published by the Department of Health and Human Services (United States) (2015 revised edition). In addition, procedures were performed to minimize the number of experimental animals and possible injuries.

### CUMS Model Development and Drug Treatment

All mice were adapted to the environment for 5 days. Based on the comprehensive scores acquired from a series of screening tests, including the open field experiment, sucrose preference test (SPT), and body weight determination, 90 mice with similar scores were selected. Ten mice were randomly selected and raised under normal conditions, which was set as the normal group. The other mice underwent CUMS procedures. The CUMS procedures were as follows: five mice were raised in one cage, and given two or three different kinds of stressors every day, and each stressor could not be repeated within three days. The stressors included: 1) food deprivation for 24 h; 2) water deprivation for 24 h; 3) inclined cage for 24 h; 4) overnight illumination; 5) restraint for 6 h; 6) wet cage for 24 h; 7) empty cage for 24 h (i.e. without cushion material); 8) stroboscopic; 9) day night reversal; (10) horizontal vibration for 30 min; 11) electric shock for 1 min; 12) foreign objects. In the first week, in order to control the mortality of mice, mild stimulation was used as far as possible. The sucrose preference test of mice was tested every week, and the specific methods of modeling were adjusted according to the sugar water preference rate and the state of mice. After 8 weeks of CUMS procedures, the CUMS model mice were randomly separated into different groups and treated with different drugs for 7 days. The groups and treatments were set as follows: normal group (0.9% saline); CUMS vehicle group (0.9% saline); D-652 at low dosage (3 g/kg/d) and high dosage (10 g/kg/d); K-984 at low dosage (3 g/kg/d) and high dosage (10 g/kg/d); K-1640 at low dosage (3 g/kg/d) and high dosage (10 g/kg/d); and fluoxetine group (positive drug group, 7.2 mg/kg/d) (Tunc-Ozcan et al., 2019; Shuto et al., 2020).

### Behavioral Evaluation

The depression-like behaviors of mice were examined using the sucrose preference test (SPT), tail suspension test (TST)and forced swimming test (FST).

The details of SPT were as follows: All mice were trained to adapt to 1% sucrose solution (w/v) 72 h before the test, where two bottles of 1% sucrose solution were placed in each cage. After 24 h, 1% sucrose in one bottle was replaced with tap water. After a 24 h adaptation period, mice were deprived of food and water for another 24 h. Then the mice were housed in individual cages with free access to two bottles containing either 100 ml of sucrose solution (1%, w/v) or 100 ml of tap water. During the test, the position of the water bottle was exchanged for 1 h to prevent the position preference. After 3 h, the weights of the consumed sucrose solution and tap water were separately recorded, and the sucrose preference was calculated using the following formula: sucrose preference = sucrose consumption/(tap water consumption + sucrose consumption) × 100%.

The details of FST and FST were as follows. In TST, individual mice were suspended in an acoustically and visually isolated chamber. Animal activities were captured using a video camera. The total time of immobility during the last 4 min in a 6-min testing period was analyzed using ANY-maze software (Stoeling Co. Ltd., United States). In FST, mice were forced to swim in a transparent glass vessel (20 cm high, 14 cm in diameter, filled with 10 cm of water at 24–26°C) placed in a cabinet. The mobilities of mice were recorded with a camera and the mice were considered immobile when they made no attempts to escape, except for the movements necessary to keep their heads above the water. The total duration of immobile time (seconds) was recorded during the last 4 min of a single 6-min test session, while the initial 2 min was applied for mouse adaptation.

### Cell Culture and Treatments

Mice microglial cell lines (BV2) were purchased from the American Type Culture Collection. The culture medium consisted of Minimum Essential Medium-Eagle (88%), fetal bovine serum (10%), penicillin/streptomycin (1%), and sodium pyruvate solution (1%). The culture medium was changed every 48 h. When the cell density reached 70%, the cells were sub-cultured. All reagents used for cell culture were purchased from Thermo Fisher Scientific (Invitrogen, Carlsbad, CA). LPS was purchased from CST (China) and 10 mg was dissolved in 10 ml sterile Phosphate-buffered saline (PBS) to prepare the stock solution (1 mg/ml). TAK-242, a TLR4 blocker, was purchased from Selleck (China) and the stock solution was 50 mM in Dimethyl sulfoxide (DMSO) (Kim et al., 2018; Gu et al., 2020). JSH-23, a transcriptional inhibitor of NF-κB, was purchased from Selleck (China) and the stock solution was 10 mM in DMSO (Cai et al., 2018; Su et al., 2020).

### ELISA Assays

After the behavioral tests, the mice were sacrificed and the hippocampal tissues were dissected. The amounts of IL-1β, IL-6, and TNF-α were determined using ELISA kits. The ELISA kits were purchased from Nanjing Jin Yibai Biological Technology Co. Ltd., and the procedures were carried out according to the manufacturer’s instructions. The detection range of the kit was 20–500 pg/ml.

### Western Blot Analysis

The experimental details of Sodium dodecyl sulfate polyacrylamide gel electrophoresis and western blot analysis can be found in our previous publication (Zhu et al., 2017). Briefly, total protein samples were loaded on 10% polyacrylamide gels and separated. The primary antibodies used were mouse polyclonal anti-TLR4 (AF7017, 1:1,000, Affinity Biosciences), rabbit polyclonal anti-NF-κB (4,764, 1:1,000, CST), rabbit polyclonal anti-p-NF-κB (5,801, 1:1,000, CST), rabbit polyclonal anti-histone H3 (4,499, 1:1,000, CST), and rabbit polyclonal anti-GAPDH (5,174, 1:1,000, CST). The secondary antibody used was anti-rabbit IgG and HRP-linked antibody (7,074, 1:5,000, CST). The bands were compared on an image analyzer, and relative quantification was performed with Image-J Digital Imaging System (Bio-Rad, Hercules, California). Gel documentation and relative quantification were performed using the Image-J Digital Imaging System.

### Real-Time Quantitative Polymerase Chain Reaction Analysis

Total RNA extraction was carried out according to the instructions listed in the TRIzol RNA extraction kit (15596-026, Thermo Fisher, United States). cDNA was acquired according to the instructions of the reverse transcription kit (AE311, Transgen Company, China). The transcriptional expression of pro-inflammatory cytokines was determined by quantitative PCR according to the instructions of the SYBR fluorescence real-time quantitative kit (AQ132, TransGen, Transgen Company, China). The primer sequences used are shown in Supplementary Table S2. Gene expression was calculated by the ΔΔCt method, and GAPDH was set as the internal reference gene.

### Immunohistochemistry

The mice were sacrificed after the behavioral tests. Entire brains were dissected and fixed in 4% paraformaldehyde (PFA) fixative solution at room temperature. Conventional dehydration and paraffin embedding were carried out afterward. Mice brain slices were dewaxed with xylene, dehydrated with an ethanol gradient, and then placed in a sodium citrate buffer solution for 15 min. The slices were cooled for 30 min and then treated with 3% hydrogen peroxide for dehydrogenation. The slices were blocked for 1 h with 5% bovine serum albumin (BSA) added with PBS-T (Triton). The primary antibody used was rabbit anti-Iba1 (19,741, 1:1,000, Wako, Japan). The secondary antibody was horseradish peroxidase labeled goat anti-rabbit IgG (H+L) antibody (1:50, Beyotime, China). DAB Horseradish Peroxidase Color Development Kit (Beyotime, China) was used to develop the color in dark for 15 min. The slices were washed with running water, dried, dehydrated and transparent, sealed with Neutral balsam (Solarbio) and photographed under microscope.

BV2 cells were washed three times with pre-cooled PBS, fixed with PBS containing 4% PFA for 30 min, and perforated with PBS permeation membrane containing 0.2% TritonX-100 for 15 min. To prevent nonspecific binding, cells were blocked with 5% BSA for 2 h at room temperature. Cells were incubated with NF-κB p65 subunit antibody (4,764, 1:1,000, CST) at 4°C overnight and then removed from the refrigerator and incubated at room temperature for 2 h. After three washes, the cells were incubated with the secondary antibody labeled with Alexa Fluor® 488 anti-rabbit antibody (1:1,000, Invitrogen) for 1 h at room temperature. DAPI (1:10,000) staining was performed for 5 min. Fluorescence images were generated by confocal microscopy.

Fluorescence images were all taken with a fluorescence microscope (X5, Zeiss, Swiss) at the corresponding excitation and emission wavelengths, and the images were processed by ZEN2012 image processing software.

### Data Analysis

Statistical tests were performed using one-way or two-way ANOVA analysis (version 13.0, SPSS, IBM Corp., Armonk, NY) followed by a Bonferroni post hoc analysis if appropriate. Before ANOVA analysis, a normal distribution test was carried out. The control group was varied in different experiments, as specified in the figure legends. Data are expressed as mean ± standard error of the mean, where n = 3–8. For behavior test, the number was set as 8. For immunohistochemistry, the number was set as 3. For western analysis, the number was set as 5. Statistically significant changes were classed as significant [*] where *p* < 0.05, highly significant [**], where *p* < 0.01.

## Results

### KXS Extracts Ameliorated Depressive-like Behaviors on CUMS-Induced Depression Mice

**FIGURE 2:**
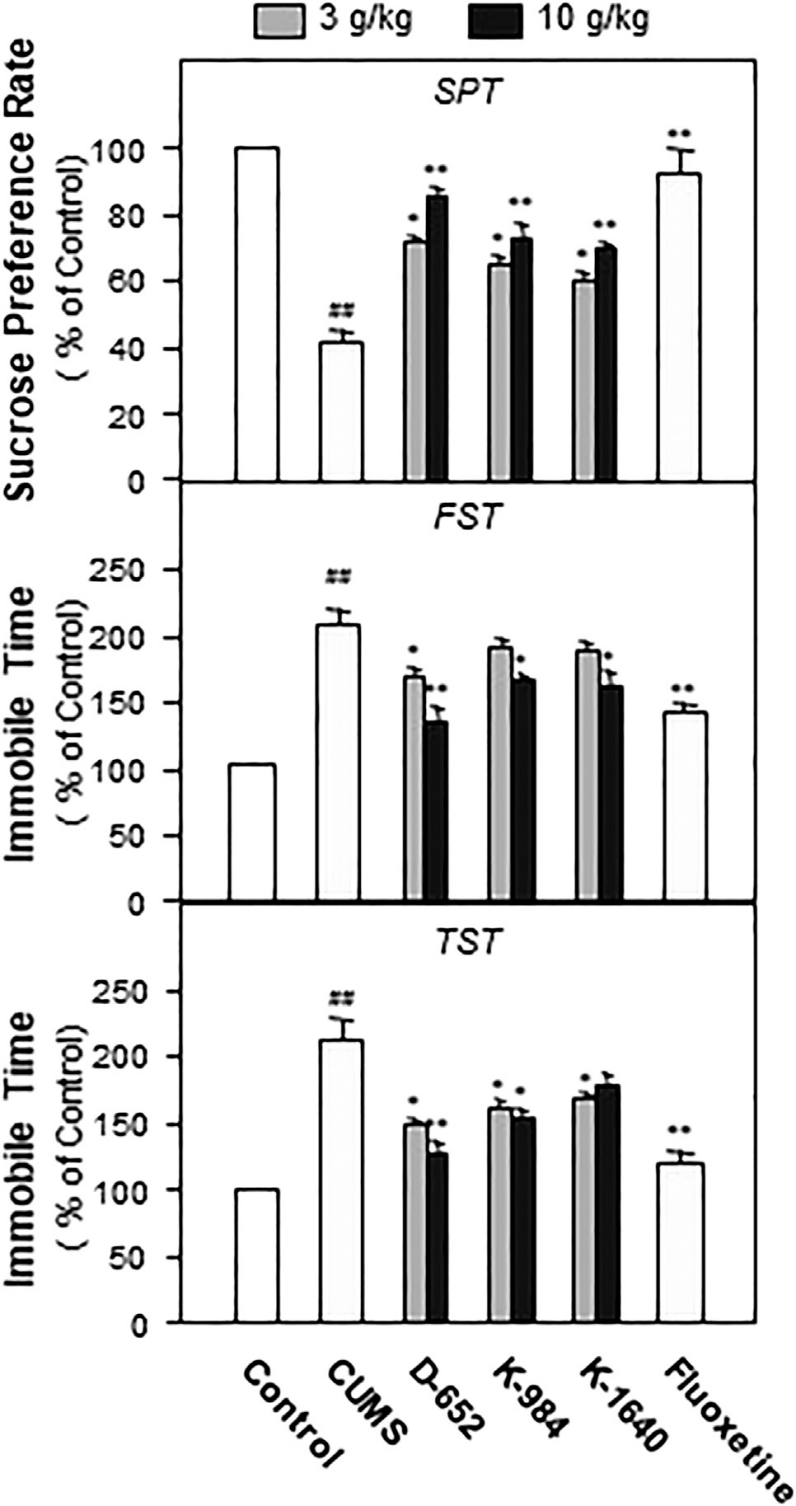
KXS extracts alleviate depressive-like behaviors in CUMS-exposed mice. The effects of KXS extracts on the sucrose consumption test (SPT), immobility time of forced swimming test (FST) and tail suspension test (TST) of CUMS-exposed mice. Values are expressed as mean ± SEM (n = 8). Comparisons between groups were carried out by a one-way ANOVA followed by a post-hoc Bonferroni test. ^##^ *p* < 0.01 (compared with the normal control group), *p < 0.05, **p < 0.01 (compared with the CUMS vehicle group).

Seven days after the administration of KXS, all mice underwent behavioral tests including the SPT, FST, and tail suspension test (TST). Statistical results showed that the consumption of sucrose in the model group was significantly lower than that in the normal control group (*p* < 0.01), and the immobile time in FST and TST was significantly higher than that in the normal group (*p* < 0.01). Compared with the CUMS vehicle group, KXS increased the sucrose preference rate and decreased the immobile time of the model animals in the FST and TST (*p* < 0.05). Among the different ratios of KXS, D-652 had the strongest effect in alleviating depression-like behaviors in animals, and its active trend was similar to that of the positive drug fluoxetine (Figure 2).

### KXS Extracts Reduced the Expressions of Inflammatory Factors in the Hippocampus of Mice

**FIGURE 3:**
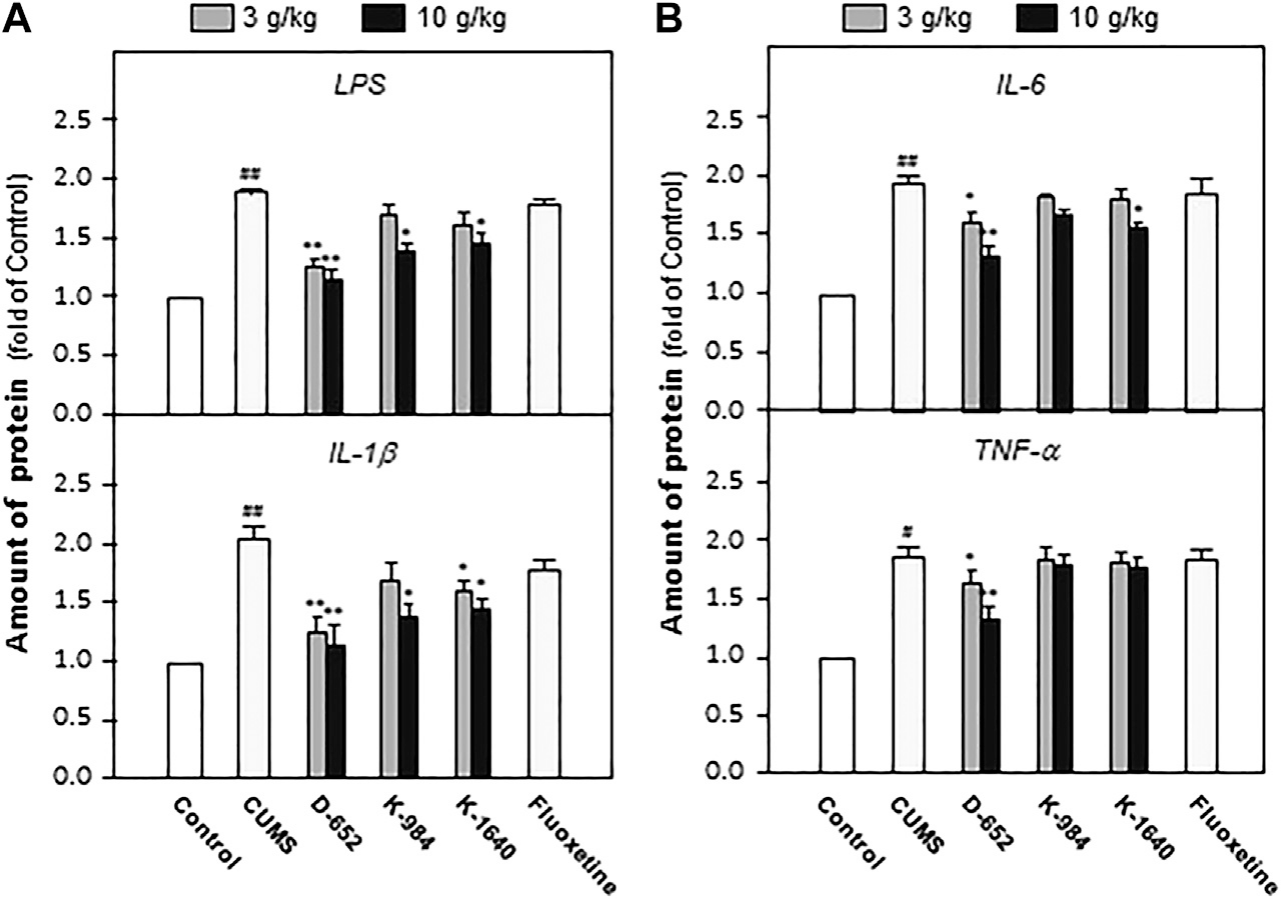
KXS extracts reduce the expression of inflammatory factors in hippocampus of CUMS-exposed mice. **(A)**: The effects of KXS on the expression of inflammatory factors LPS and IL-1β in hippocampus of CUMS-exposed mice; **(B)**: The effects of KXS on the expression of inflammatory factors IL-6 and TNF-α in hippocampus of CUMS-exposed mice. Values are expressed as the percentage of the control group (normal mice), as Mean ± SEM (n = 8). Comparisons between groups were carried out by a one-way ANOVA followed by a post-hoc Bonferroni test. ^#^ *p* < 0.05, ^##^ *p* < 0.01 (compared with control group), *p < 0.05, **p < 0.01 (compared with CUMS vehicle group).

After behavioral tests, the groups of mice were sacrificed, and the hippocampal tissues were isolated. The levels of LPS, IL-1β, IL-6, and TNF-α were measured using an ELISA kit. The experimental results showed that the levels of LPS, IL-1β, IL-6, and TNF-α in the hippocampus of CUMS model mice were significantly upregulated compared with those of the normal control group (*p* < 0.01). KXS significantly downregulated the expression of LPS, IL-1β, IL-6, and TNF-α in the hippocampus of CUMS model animals compared with the untreated CUMS vehicle group (*p* < 0.05). In line with the behavioral tests, D-652 treatment at high dosage showed the strongest active trend (Figure 3).

### Effect of KXS Extracts on Microglia in Mouse hippocampus

**FIGURE 4:**
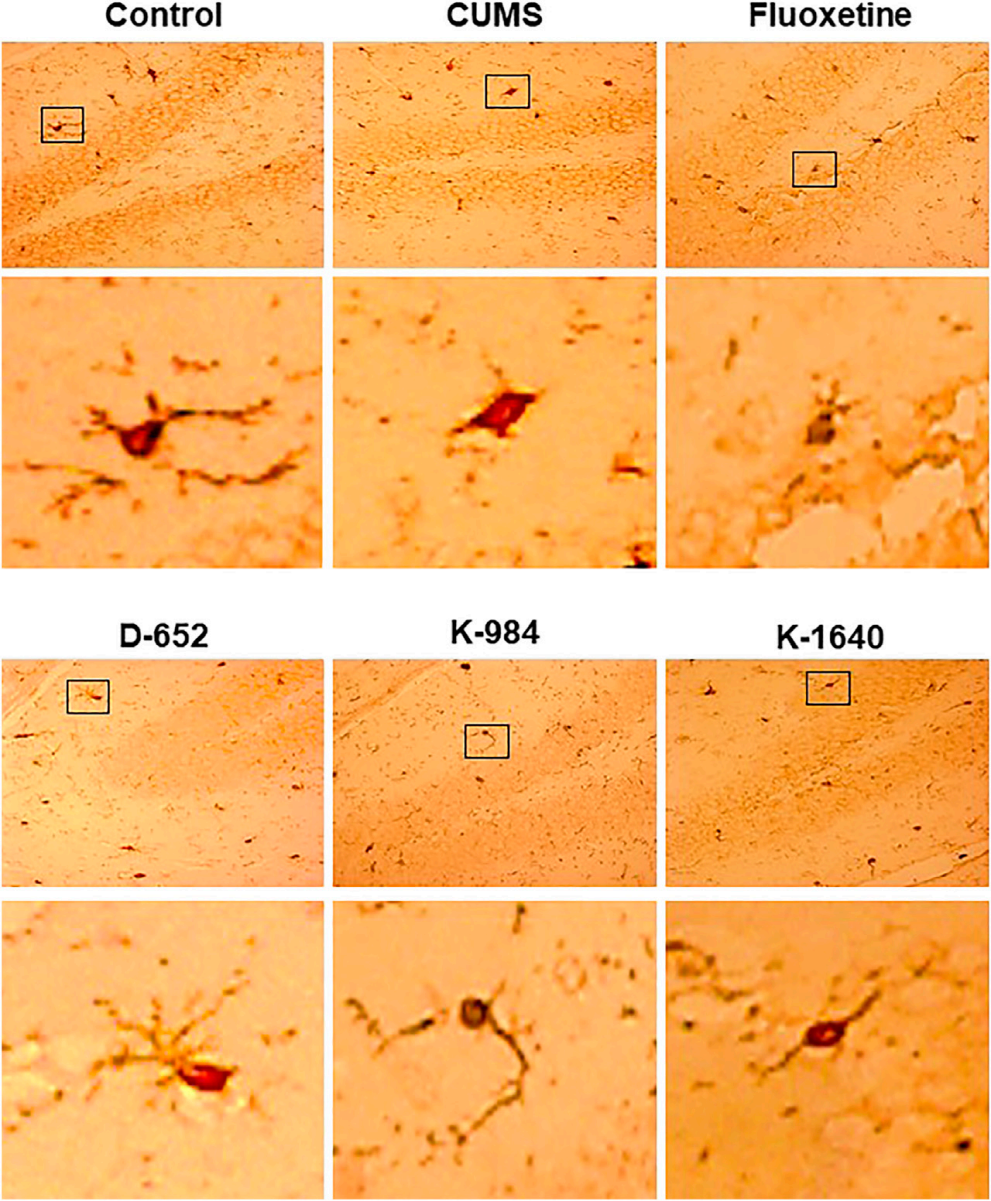
KXS extracts affect the morphology of microglia in mice. The morphologies of microglia in dentate gyrus of mice in normal control group, CUMS vehicle group, D-652 administration group, K-984 administration group, K-1640 administration group, and fluoxetine administration group, were determined by immunoperoxide method. The doses of D-652 administration group, K-984 administration group, K-1640 administration group were set as 10 g/kg/d (n = 3). Correspondingly, the larger images of microglia in normal control group, CUMS vehicle group, D-652 administration group, K-984 administration group, K-1640 administration group, and fluoxetine administration group were displayed.

After the animals were sacrificed, the entire brains of the mice were isolated. Morphological changes in microglia in hippocampal tissues were observed by fluorescence microscopy. The results showed that the microglia in the hippocampus of mice in the normal control group were in a branching state, suggesting that they were inactivated. Meanwhile, the microglia in the hippocampus of mice in the CUMS vehicle group were in a circular state, suggesting that they were activated. The morphology of microglia in the KXS-treated group was similar to that in the normal group, indicating that their activation status could be inhibited by KXS treatment, especially in the D-652 treatment group. The positive drug fluoxetine group had no obvious inhibitory effect on microglial activation (Figure 4).

### KXS Extracts Decreased the Expressions of TLR4 Receptor and Inhibited NF-κB Phosphorylation in the Hippocampus of Mice

**FIGURE 5:**
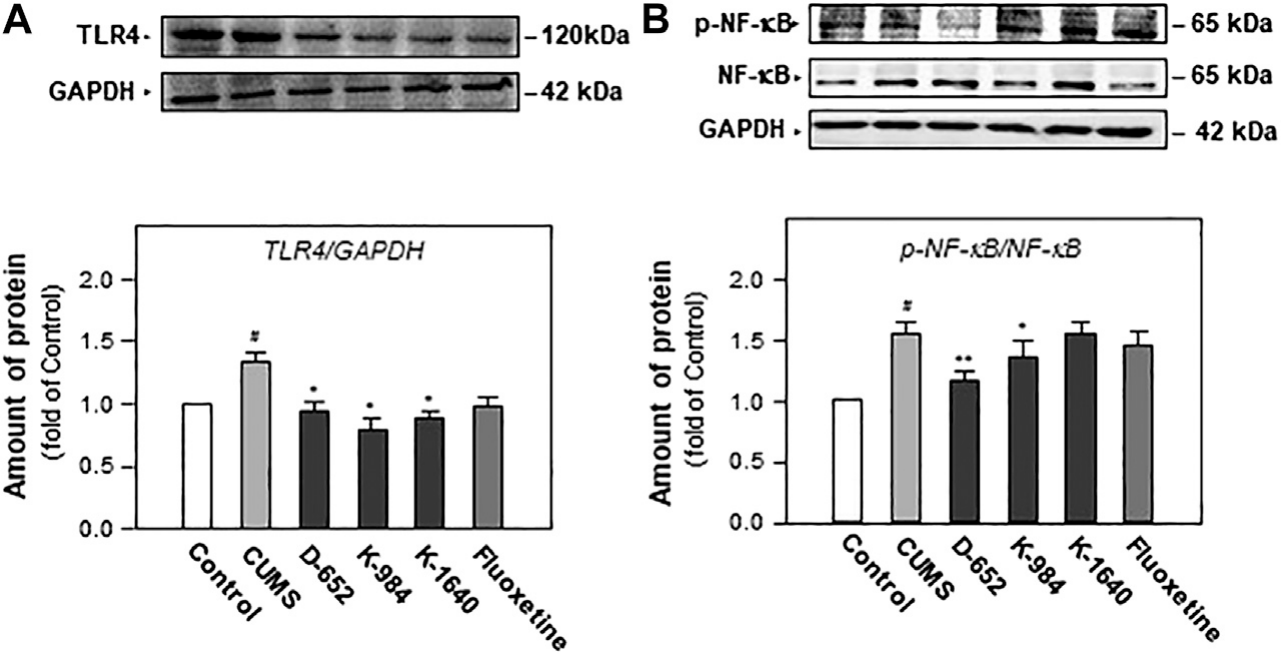
KXS extracts regulate the expression of proteins of TLR4/NF-κB signaling pathway in the hippocampus of CUMS-exposed mice. **(A)**: The effects of KXS extract (10 g/kg/d) on TLR4 protein expression in the hippocampus of CUMS-exposed mice; **(B)**: The effects of KXS extracts on expressions of p-NF-κB and NF-κB in the hippocampus of CUMS-exposed mice; Values are expressed as the percentage of the control group (normal mice), as Mean ± SEM (n = 8). Comparisons between groups were carried out by a one-way ANOVA followed by a post-hoc Bonferroni test. ^#^ *p* < 0.05, (compared with control group), *p < 0.05, **p < 0.01 (compared with CUMS group).

After behavioral tests, the expression of TLR4 and NF-κB phosphorylation in the hippocampus of mice, two proteins closely related to the NF-κB signaling pathway, were studied. As shown in Figures 5A,B, the expression of TLR4 and the phosphorylation level of NF-κB protein increased in the hippocampus of CUMS vehicle group mice compared with those of the normal control group (*p* < 0.05). These phenomena suggest that chronic unpredictable stress promotes NF-κB protein entry into the nucleus by activating the phosphorylation of the NF-κB protein in the hippocampus of mice. Compared with the CUMS vehicle group, D-652 decreased the expression of TLR4 (*p* < 0.05) and inhibited the phosphorylation level of the NF-κB protein (*p* < 0.01), thus inhibiting the expression of inflammatory factors in the hippocampus of CUMS model mice.

### KXS Extracts Decreased the Expressions of Pro-Inflammatory Cytokines by Regulating the TLR4/IKK/NF-κB Signaling Pathway and Inhibiting Nuclear Translocation of NF-κB on BV2 Cells

**FIGURE 6:**
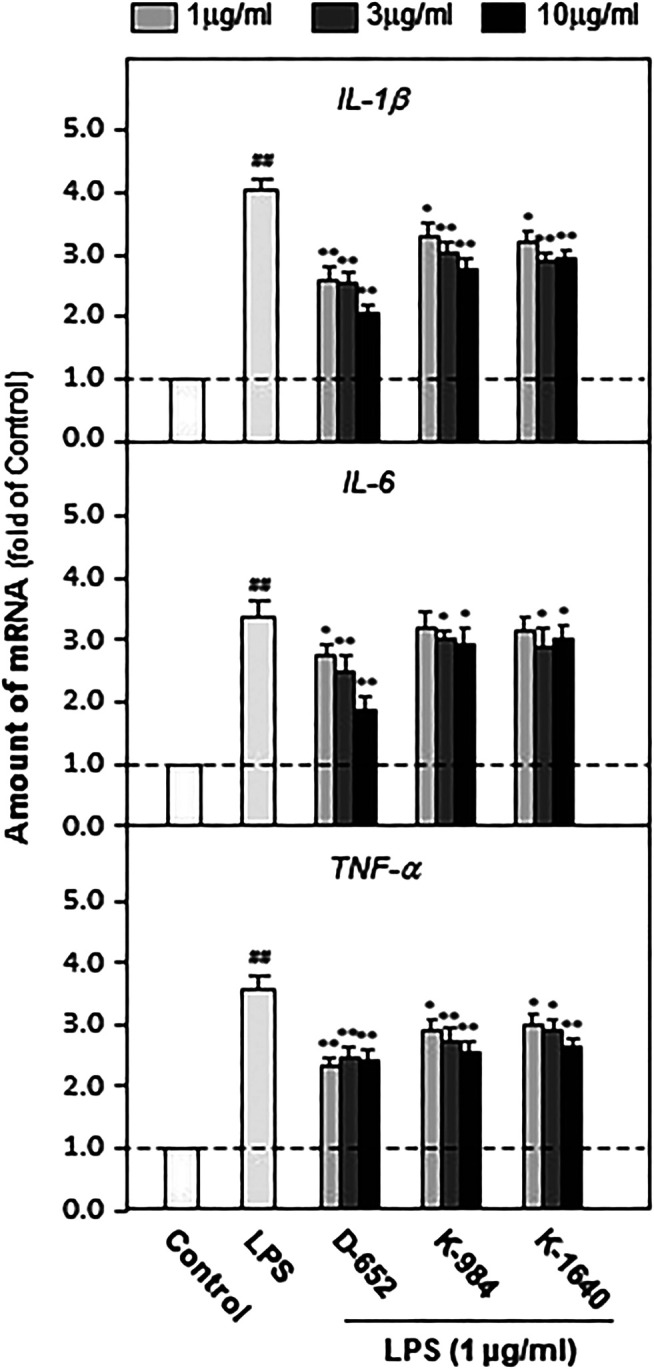
KXS extracts decrease the expressions of pro-inflammatory factors in LPS-treated BV2 cells. BV2 cells were treated with LPS (1 μg/ml) and different compatibility ratios of KXS extracts simultaneously. The expressions of IL-1β, IL-6, TNF-α were determined by qPCR. Values are expressed as the percentage of the control group (no drug treatment) as Mean ± SEM (n = 5). Comparisons between groups were carried out by a one-way ANOVA followed by a post-hoc Bonferroni test. ^##^ *p* < 0.01 (compared with control group), *p < 0.05, **p < 0.01 (compared with LPS treatment group).

We constructed an *in vitro* cell model mimicking central nervous system inflammation by treating the microglial cell line (BV2) with LPS to stimulate pro-inflammatory cytokine expression. Subsequently, the effects of KXS on the expression levels of IL-1β, IL-6, and TNF-α were evaluated. As shown in Figure 6, LPS at 1 μg/ml significantly promoted the expression of inflammatory factors in BV2 cells compared with that in the untreated control group (*p* < 0.01), and KXS extracts significantly inhibited the expression of pro-inflammatory factors in BV2 cells stimulated by LPS (*p* < 0.01). In addition, KXS extracts had no effect on the expression of inflammatory factors in BV2 cells (data were not shown). Based on the animal and cell experiments, the D-652 compatibility ratio exerting the best active trend in improving depression-like behaviors and suppressing pro-inflammatory cytokine expression was selected for further study on the mechanism of KXS pro-inflammatory cytokine expression.

**FIGURE 7:**
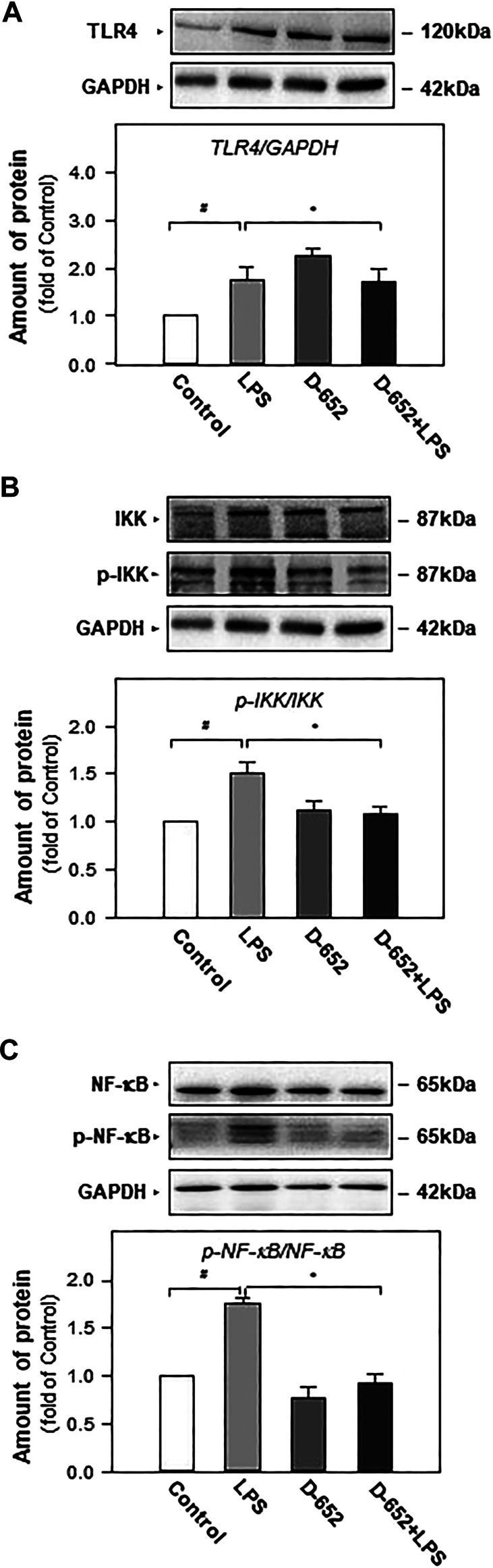
KXS extracts regulate the expression of proteins of TLR4/NF-κB signaling pathway in LPS-treated BV2 cells. **(A)**: The effect of KXS extracts D-652 on the TLR4 expression in LPS-treated BV2 cells at 2 h; **(B)**: The effect of KXS extracts D-652 on the proteins of *p*-IKK and IKK in LPS-treated BV2 cells at 2 h; **(C)**: The effect of KXS extracts D-652 on the proteins of p- NF-κB and NF-κB in LPS-treated BV2 cells at 2 h; Values are expressed as the percentage of the control group (no drug treatment) as Mean ± SEM (n = 5). Comparisons between groups were carried out by a one-way ANOVA followed by a post-hoc Bonferroni test. ^##^ *p* < 0.01 (compared with control group), *p < 0.05 (compared with LPS treatment group).

**FIGURE 8:**
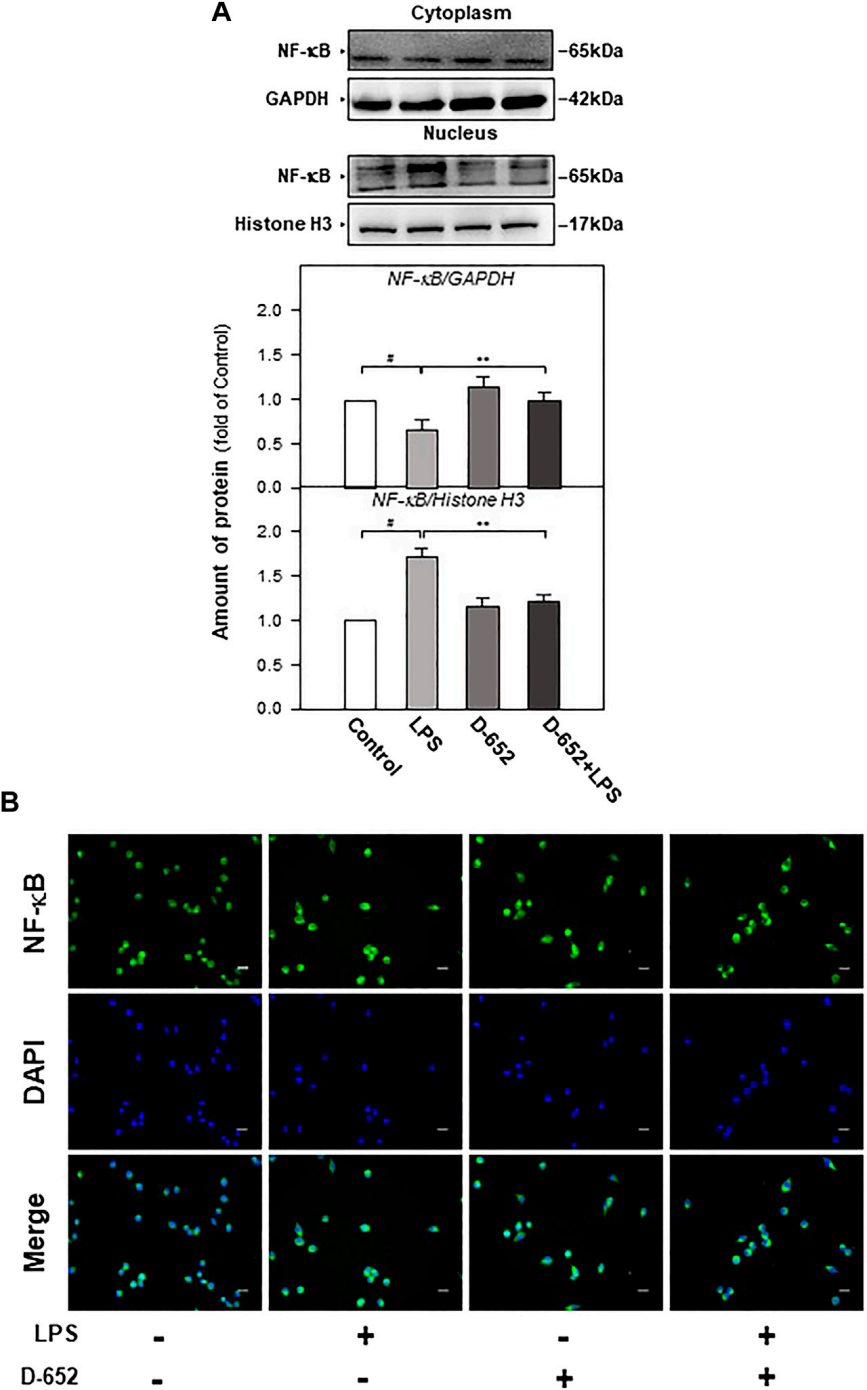
KXS extracts regulate the nucleus translocation of the NF-κB protein in LPS-treated BV2 cells. **(A)**: The effect of KXS extracts D-652 on the expressions of NF-κB in nucleus and cytoplasm in LPS-treated BV2 cells at 2 h; **(B)**: The immunofluorescent images of the NF-κB nucleus translocation were observed with fluorescent microscopy in LPS-treated BV2 cells at 2 h; Green, NF-κB; blue, DAPI; scale bar = 5 μm. Values are expressed as the percentage of the control group (no drug treatment) as Mean ± SEM (n = 5). Comparisons between groups were carried out by a one-way ANOVA followed by a post-hoc Bonferroni test. ^##^ *p* < 0.01 (compared with control group), *p < 0.05 (compared with LPS treatment group).

When BV2 cells were treated with LPS, the expression of TLR4 was significantly increased compared to that in the normal control group. This increasing trend was significantly reversed by treatment with D-652 (Figure 7A). A similar active trend was found in both the IKK phosphorylation levels and NF-κB phosphorylation levels (Figures 7B,C). As shown in Figures 8A,B, we further found that LPS treatment significantly promoted the nuclear translocation of NF-κB (*p* < 0.01) and D-652 significantly inhibited the nuclear translocation of NF-κB (*p* < 0.01).

**FIGURE 9:**
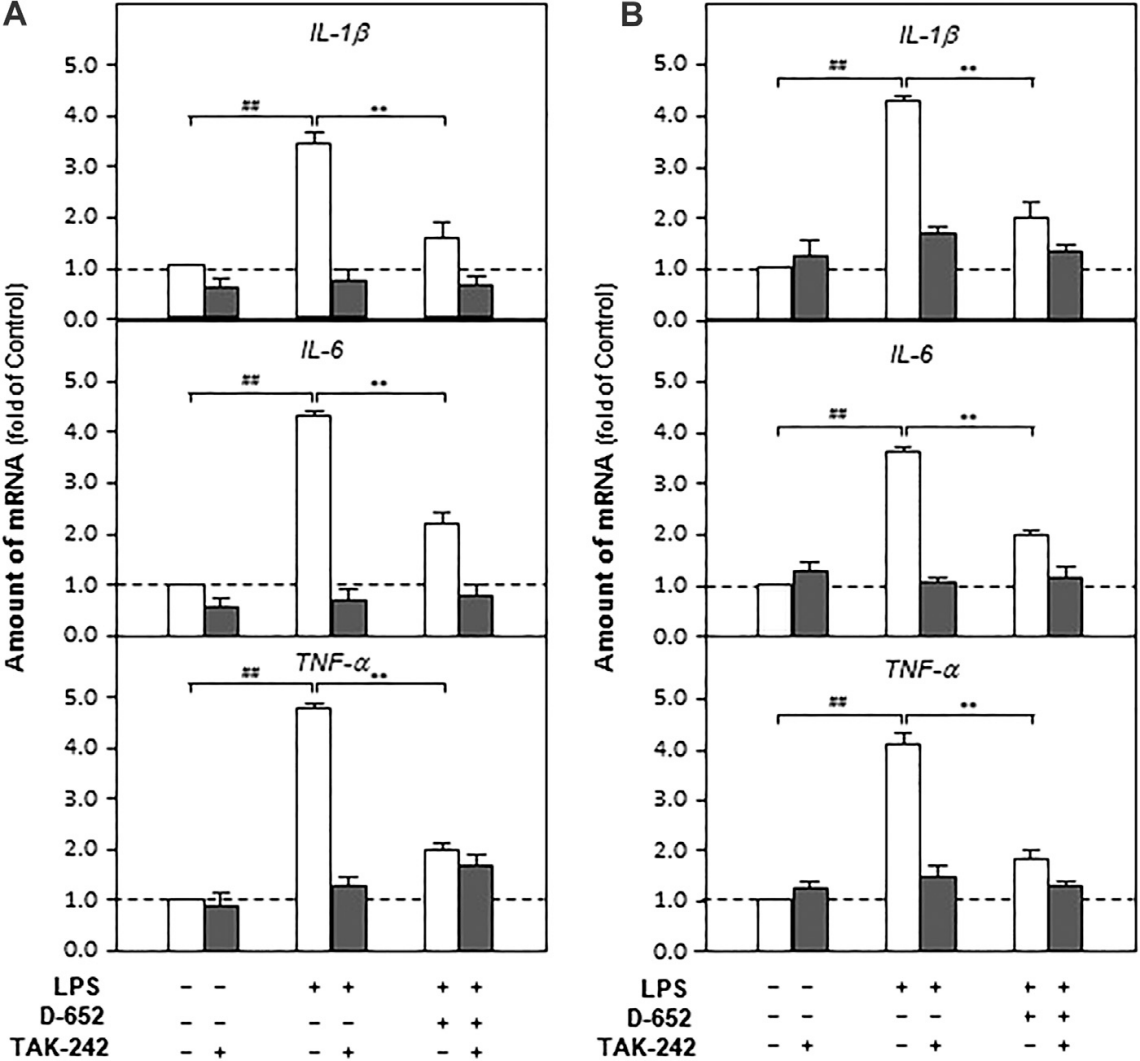
Effect of KXS extracts on the expressions of pro-inflammatory factors in LPS-treated BV2 cells after administration of TAK-242 and JSH-23. **(A)**: Effect of KXS extracts on the expressions of pro-inflammatory factors in LPS-treated BV2 cells after administration of TAK-242. **(B)**: Effect of KXS extracts on the expressions of pro-inflammatory factors in LPS-treated BV2 cells after administration of JSH-23. Values are expressed as the percentage of the control group (no drug treatment) as Mean ± SEM (n = 5). Comparisons between groups were carried out by a two-way ANOVA followed by a post-hoc Bonferroni test. *p < 0.05.

Furthermore, TAK-242 (the inhibitor of TLR4) and JSH-23 (an inhibitor of NF-κB nuclear translocation) were simultaneously treated with D-652 on BV2 cells to verify whether KXS simultaneously decreased pro-inflammatory cytokine expression by regulating the TLR4/IKK/NF-κB signaling pathway. As shown in Figures 9A,B, single D-652 treatment and single TAK-242 treatment both significantly decreased the expression of pro-inflammatory cytokines in LPS-treated BV2 cells. However, the combined treatment of TAK-242 and D-652 exerted no significant effects on the expression of pro-inflammatory cytokines compared with single D-652 or TAK-242 treatment groups. Therefore, the downregulated expression of pro-inflammatory cytokines of D-652 and TAK-242 co-treatment was exerted mainly by TAK-242 instead of D-652. In other words, the downregulation effect of D-652 on pro-inflammatory cytokine expression was attenuated. A similar effect was also found in the combined treatment of JSH-23 and D-652.

**FIGURE 10:**
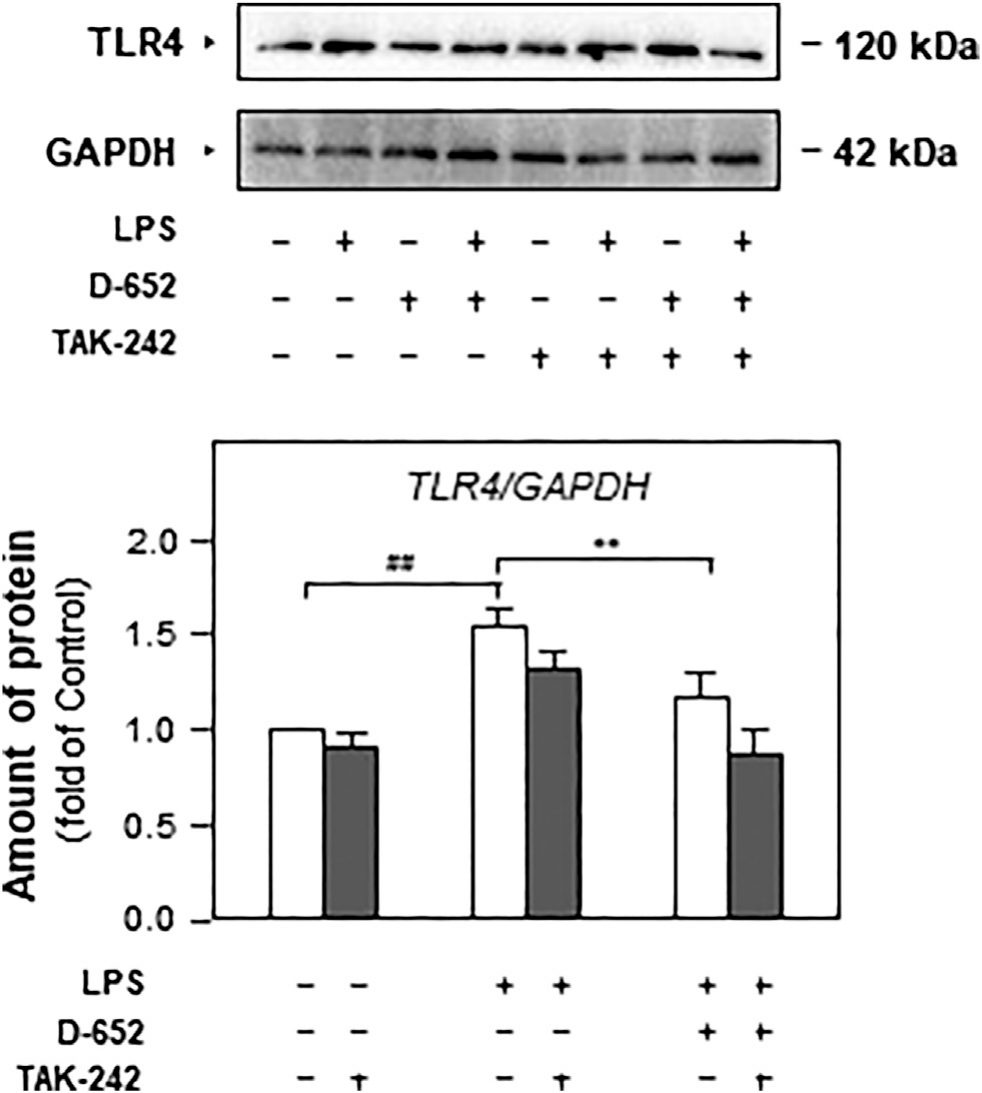
Effect of KXS extracts on the expressions of TLR4 in LPS-treated BV2 cells after administration of TAK-242. Effect of KXS extracts D-652 on the TLR4 expression in LPS-treated BV2 cells after administration of TAK-242 at 2 h; Values are expressed as the percentage of the control group (no drug treatment) as Mean ± SEM (n = 5). Comparisons between groups were carried out by a two-way ANOVA followed by a post-hoc Bonferroni test. *p < 0.05.

**FIGURE 11:**
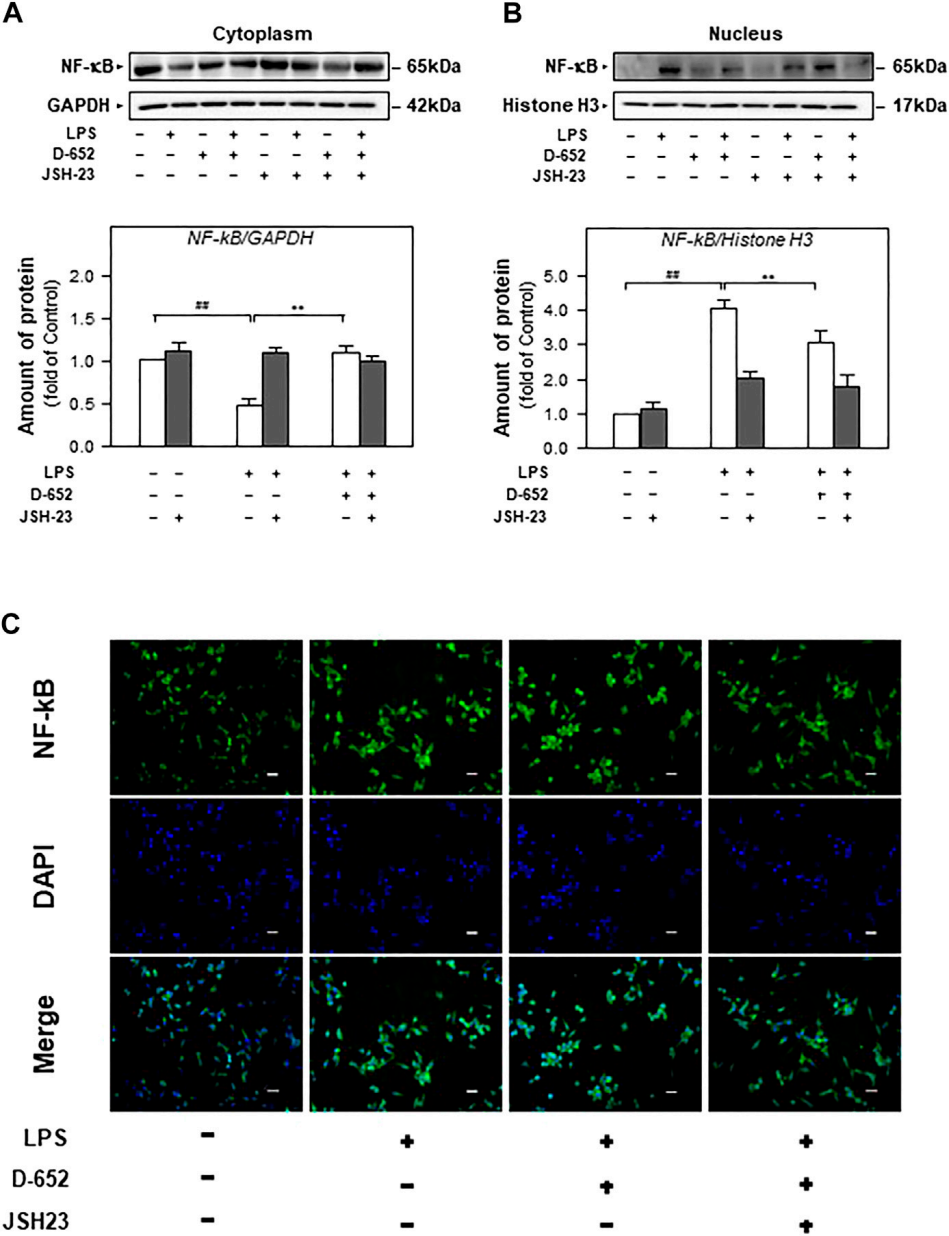
Effect of KXS extracts on the nucleus translocation of the NF-κB protein in LPS-treated BV2 cells after administration of JSH-23. **(A)**: Effect of KXS extracts D-652 on the NF-κB expression in cytoplasm of LPS-treated BV2 cells after administration of JSH-23 at 2 h; **(B)**: Effect of KXS extracts D-652 on the NF-κB expression in nucleus of LPS-treated BV2 cells after administration of JSH-23 at 2 h; **(C)**: The immunofluorescent images of the NF-κB nucleus translocation were observed with fluorescent microscopy in LPS-treated BV2 cells after administration of JSH-23 of D-652 at 2 h; Green, NF-κB; blue, DAPI; scale bar = 5 μm. Values are expressed as the percentage of the control group (no drug treatment) as Mean ± SEM (n = 5). Comparisons between groups were carried out by a two-way ANOVA followed by a post-hoc Bonferroni test. ^##^ *p* < 0.01 (compared with control group), *p < 0.05 (compared with LPS treatment group).

We also evaluated the effect of TAK-242 on TLR4 expression in D-652 treated LPS-exposed BV2 cells. As shown in Figure 10, TAK-242 significantly inhibited LPS-induced increase in TLR4 expression. The downregulation effect of D-652 on TLR4 was also attenuated by simultaneous treatment with TAK-242. Similarly, the inhibition of D-652 on nucleus translocation of NF-κB was also attenuated by JSH-23 (Figures 11A–C).

## Discussion

In the current study, we found that KXS significantly alleviated depression-like behaviors, decreased the LPS and pro-inflammation factors, and suppressed the activation of microglia in the hippocampus of CUMS-exposed depression-like mice. In BV2 microglia cell lines, KXS extracts significantly decreased the expression of IL-1β, IL−2, and ΤΝF-α induced by LPS and inhibited TLR4/IKK/NF-κB signaling pathways, which might be indispensable in the antidepressant effect of KXS.

At present, central neuronal inflammation has been recognized to play an important role in the pathogenesis and development of MDD. Clinical findings showed that the serum levels of inflammatory cytokines IL-1β, IL-6, and TNF-α in patients with MDD were significantly higher than those in normal people. Microglia are the main cells responsible for inflammation in the brain. Animal studies have also confirmed that sustained chronic stress stimulates the HPA axis and activates microglia to secrete inflammatory cytokines, which impairs the survival and development of neurons. The impairment of neurons leads to decreased CX3CL1 release, which further activates microglia to induce pro-inflammatory cytokine release, which further aggravates neuronal inflammation. In addition, neuronal inflammation also stimulates the tryptophan-kynurenic acid metabolic pathway, which reduces serotonin synthesis and leads to insufficient supply of serotonin. In neuronal inflammation, the activated NF-κB signaling pathway is the principal pathway that promotes the expression of pro-inflammatory cytokines in microglia, and LPS is an important inducer of this signaling pathway. LPS acts on TLR4 receptors in microglia, promotes NF-κB in the cytoplasm to enter the nucleus, exerts transcriptional regulation, and promotes the synthesis and secretion of pro-inflammatory cytokines. Therefore, relieving neuronal inflammation and inhibiting activated TLR4/NF-κB should be included in new-generation antidepressant development (Yirmiya et al., 2015; Zhang et al., 2017; Deczkowska et al., 2018).

At present, the available antidepressants in clinics are monoamine neurotransmitter reuptake inhibitors. No antidepressant has been developed to suppress central nervous system inflammation. Studies have shown that the serotonin reuptake inhibitors fluoxetine and escitalopram can significantly reduce the expression of IL-1β, IL-6, TNF-α, and inducible nitric oxide synthase in BV2 cells and primary microglia induced by the combination of LPS and interferon-γ (Su et al., 2015; Xiaoling et al., 2018). The serotonin and norepinephrine reuptake inhibitors venlafaxine can downregulate the expression of inflammatory factors IL-1β, IL-6, and TNF-α induced by morphine in mice, thus exerting the effects of anti-neuroinflammation. However, these antidepressants are ineffective in 40% of patients with MDD and cause side effects such as gastrointestinal discomfort and sexual dysfunction. In addition, in view of the complex pathological networks of MDD, single compounds with limited action targets may not be suitable for this sophisticated disease, while Chinese medicine formulae composed of multiple components and action targets might be an effective alternative therapy for MDD (Degner et al., 2004).

Traditional Chinese medicine has rich experience in the prevention and treatment of depression. At present, the number of compound Chinese medicine in the stage of new drug development is increasing year by year. Among them, Yueju pill, Ganmai Dazao decoction, Chaihu Shugan powder, Sini Powder, KXS and so on are representative Chinese herbal compound. Animal experiments showed that the petroleum ether extract of Yueju pill could alleviate the depressive behavior of mice by promoting the release of BDNF and the activation of TrkB (Xue et al., 2013). Clinical studies have shown that Ganmai Dazao decoction can exert antidepressant effect by affecting the content of monoamine neurotransmitters in brain regions (Ma et al., 2014). Animal experiments show that Chaihu Shugan powder can alleviate the depressive behavior of rats by increasing the expression of BDNF and TrkB in hippocampus, amygdala and frontal lobe (Zhan et al., 2004). Animal experiments show that Sinisan can reduce the increase of serum corticosterone and plasma ACTH by inhibiting the over activation of HPA axis, thus exerting the antidepressant effect (Wei et al., 2016). KXS is an effective treatment for mild and moderate depression. Studies have shown that KXS can significantly alleviate the depression like behavior of CUMS mice, and play an antidepressant role by promoting the release of neurotransmitters and neurotrophic factors in the brain of mice and inhibiting the excessive activation of HPA axis. And KXS is rich in anti-inflammatory active ingredients, such as ginsenoside and Ginsenoside Rg3, which can inhibit the activation of HPA axis in chronic stress depression rats, reduce the levels of IL-1β, IL-6 and TNF-α in hippocampus, and improve the level of 5-hydroxytryptamine in hippocampus by inhibiting the metabolism of tryptophan to kynuric acid. Ginseng, as the main component of KXS, can significantly alleviate the central nervous system inflammation, which also reveals the application prospect of KXS in regulating central nervous system inflammation and alleviating depression. KXS has extensive research value in relieving depression because of its multi-component, multi-target and multi-channel pharmacological action.

KXS is one of the most frequently applied formulae for treating MDD in Chinese medicine clinics. The components contained in KXS have been confirmed to regulate neuronal inflammation. The total ginsenoside and ginsenoside Rg3 in Radix Ginseng can inhibit HPA axis activation in rats with chronic stress-induced depression. They reduce the levels of IL-1β, IL-6, and TNF-α in the hippocampus, and increase the level of 5-HT in the hippocampus by inhibiting the metabolism of tryptophan into canine uric acid (Kang et al., 2011; Kang et al., 2017; Xu et al., 2018). In the current study, we also found that D-652, with a higher content of Ginseng Radix, exerted the strongest antidepressant effect. Alfa-asarone in Rhizoma Acori Tatarinowii extract can inhibit the expression of inflammatory factors, regulate NF-κB transcription levels, and block the release of inflammatory factors in senile rats and primary cultured microglia by blocking the ubiquitination of IκB-α and β kinases, thereby playing a role in resisting neuroinflammation. It has been reported that β-asarone, also an active ingredient of Rhizoma Acori Tatarinowii, can improve the damage of cognitive and synaptic plasticity by regulating the excessive release of pro-inflammatory cytokines and the activation of microglia (Lim et al., 2014; Liu et al., 2017). Although there are no direct reports of compounds from Polygalae Radix and Poria on the regulation of central neuronal inflammation, senegenin in Polygalae Radix et Rhizoma can reduce the symptoms of colitis by regulating IFN-γ and IL-4 (Hong et al., 2002; Cheong et al., 2011). Total triterpenoids of Poria can fight against acute inflammation in mice with auricle swelling and an increase in abdominal cavity capillary permeability caused by xylene. Poria neonate C was also found to inhibit the expression of INOS and COX-2 by downregulating the expression of NF-κB protein to play an anti-inflammatory role (Ríos, 2011). Therefore, the antidepressant effect of KXS might be due to the synergistic effect of the multiple compounds of the four herbs.

In addition to KXS, some Chinese medicine formulae also exerted antidepressant effects by regulating neuronal inflammation. The Ban-Xia-Hou-Pu decoction plays an antidepressant role by inhibiting the activation of NLRP3 inflammasomes and the production of IL-1β in the liver, hypothalamus, hippocampus, or prefrontal cortex of CUMS-induced depression rats (Jia et al., 2017). Si-Ni-San reduces the release of inflammatory factors IL-1β, IL-6, IL-2, and TNF-α in the peripheral fluid of patients with post-stroke depression and regulates the over-activation of the HPA axis, thereby exerting an antidepressant effect (Guo et al., 2009; Li et al., 2013). Chang-Yu-Xiao-Yao-San can reduce the expression of inflammatory factors IL-6, IL-8, and TNF-α in the serum of patients with chronic hepatitis B combined with depression, and relieve the symptoms of depression in patients. Animal experimental results also show that Chang-Yu-Xiao-Yao-San inhibits an over-expression of serum IL-6, IL-8, and TNF-α in LPS-induced depression model rats and increased the content of hippocampal 5-HT in rats (Qi et al., 2013; Jing et al., 2015). However, the above-mentioned study on the inhibition of central nervous inflammation by traditional Chinese medicine compounds is often limited to the determination of inflammatory factors, and there is a lack of in-depth research on the mechanism. In addition, the differences in the antidepressant regulation networks between these formulae are worth elucidating.

Besides, we found that D-652 increased TLR4 expressions in BV2 cells alone while decreased TLR4 expressions both in LPS treated BV2 cells and CUMS depressive mice. Interestingly, this contradictory phenomenon is also reported in Radix Ginseng. *In vitro*, ginsenosides is reported to activate phagocytosis on macrophage RAW264.7 cells through regulating TLR2/4 and NF-κB signaling pathways and the enhancement of TLR4 expression is also found (Xu et al., 2018; Gao et al., 2020). *In vivo*, Radix Ginseng is also reported to down-regulate the TLR4 expressions in disease-like animal models like cerebral Ischemia rats (Cheng et al., 2019; Li et al., 2020). These findings support that Radix Ginseng exert bi-directional function in regulating immunity by enhancing phagocytosis in normal condition while inhibiting abnormal stimulation of phagocytes in pathological condition. Since Radix Ginseng is the major herb of D-652, we hypothesize that Radix Ginseng might contribute to the enhanced expressions of TLR4 in BV2 cells. However, we will further investigate the details in the future. Besides, we will further study the functional material basis and the in-depth mechanism of KXS suppression of central neuronal inflammation, which will provide more scientific evidence for the development of new antidepressants or alternative therapies from this Chinese medicine formula.

## Conclusion

KXS extracts play an antidepressant role by improving depression-like behaviors on CUMS-exposed mice. This effect may be related to the inhibition of the release of inflammatory factors in microglia of hippocampus and regulation of TLR4/IKK/NF-κB signaling pathway on BV2 cells. This study will be helpful for the antidepressant alternative therapies or drug developments (Zhu et al., 2012).

## Data Availability

The raw data supporting the conclusions of this article will be made available by the authors, without undue reservation.
