# Supplementary material for: Chinese Medicine Formula Kai-Xin-San Ameliorates Neuronal Inflammation of CUMS-Induced Depression-like Mice and Reduces the Expressions of Inflammatory Factors via Inhibiting TLR4/IKK/NF-κB Pathways on BV2 Cells
Source: Front Pharmacol. 2021 Mar 11;12:626949. doi: 10.3389/fphar.2021.626949 (PMC8006317; doi:10.3389/fphar.2021.626949)
Supplement: Supplementary file 1 [file datasheet1.docx]

**Supplementary Table 1.** Historical record of KXS series formulae.

| Notation | Record | Ratio | | | |
| --- | --- | --- | --- | --- | --- |
|  |  | GR | PR | ATR | PO |
| D-652 | *Beiji Qianjin Yaofang* | 3 | 2 | 2 | 3 |
| K-984 | *Yixin Fang* | 1 | 1 | 1 | 2 |
| K-1640 | *Jinyue Quanshu* | 1 | 1 | 4 | 8 |

**Supplementary Table 2 Criteria for standardized KXS series formulae**

| **Chemical** **marker** | **D-652** | **K-984** | **K-1640** |
| --- | --- | --- | --- |
| Ginsenoside Rb_1_ | 205.7 ± 3.5 | 192.6 ± 9.2 | 91.5 ± 8.8^a^ |
| Ginsenoside Rd | 86.2 ± 4.6 | 75.3± 8.9 | 43.2 ± 9.1 |
| Ginsenoside Re | 197.2 ± 8.2 | 164.9 ± 3.9 | 75.3 ± 8.3 |
| Ginsenoside Rg_1_ | 201.3 ± 1.5 | 172.3 ± 8.8 | 110.2 ± 8.1 |
| 3, 6’-disinapoyl sucrose | 242.6 ± 3.9 | 287.6 ± 9.7 | 159.8 ± 7.8 |
| α-Asarone | 40.5± 3.7 | 57.6 ± 6.7 | 71.2 ± 8.3 |
| β-Asarone | 356.18 ± 2.8 | 467.8 ± 8.9 | 587.2 ± 14.1 |
| Pachymic acid | 5.5e^-3^ ± 0.3 e^-3^ | 24.9 e^-3^ ± 3.9 e^-3^ | 22.3 e^-3^ ± 4.9 e^-3^ |

^a^ Values were expressed in mg/100g of dried powder of KXS, Mean ± SEM, n=3.

**Supplementary Table 3 Primer sequences**

| **Primer** | **Sequence (5’-3’)** | **Gene bank number** |
| --- | --- | --- |
| IL-1β-S | TGC CAC CTT TTG ACA GTG ATG | NM_008361.4 |
| IL-1β-AS | TGATACTGCCTGCCTGAAGC |  |
| IL-6-S | TCG TGG AAA TGA GAA AAG AGT TG | NM_012589.2 |
| IL-6-AS | CCA GGT AGC TAT GGT ACT CC |  |
| TNF-α-S | CAC GTC GTA GCA AAC CAC C | AE013599.5 |
| TNF-α-AS | TGG GTG AGG AGC ACG TAG |  |
| β-actin-S | TAC GAG GGC TAT GCT CTCC | AE014298.5 |
| β-actin-AS | CAC GCA CGA TTT CCC TCTC |  |
