# Supplementary figures and images for: Chinese Medicine Formula Kai-Xin-San Ameliorates Neuronal Inflammation of CUMS-Induced Depression-like Mice and Reduces the Expressions of Inflammatory Factors via Inhibiting TLR4/IKK/NF-κB Pathways on BV2 Cells
Source: Front Pharmacol. 2021 Mar 11;12:626949. doi: 10.3389/fphar.2021.626949 (PMC8006317; doi:10.3389/fphar.2021.626949)

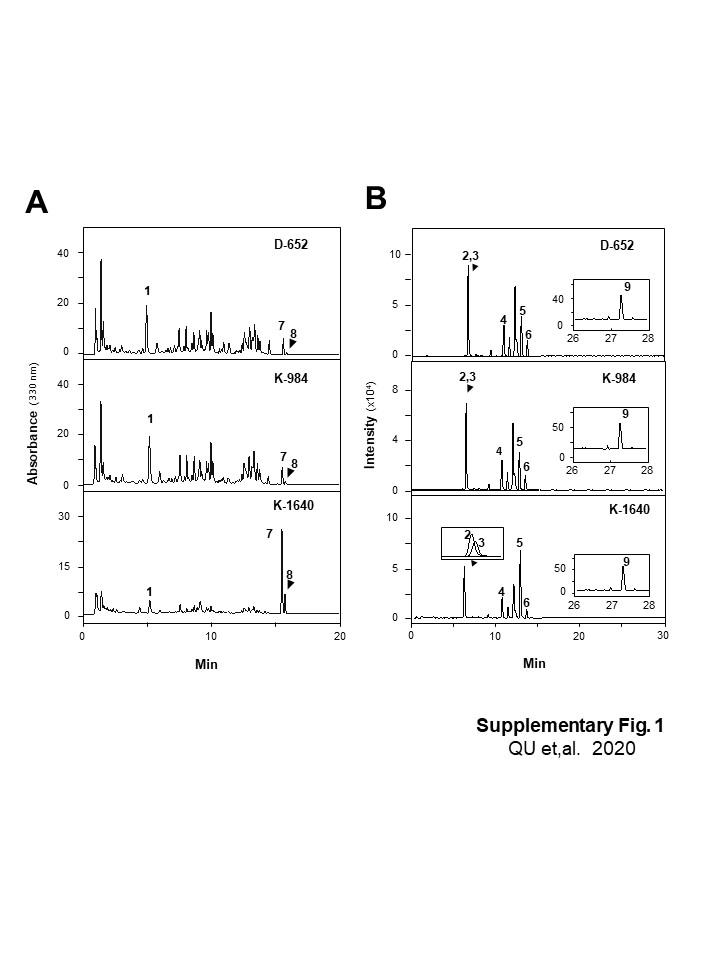

Supplement: Supplementary file 2 [file image1.jpeg]
